# Does Long-Term Administration of a Beta-Blocker (Timolol) Induce Fibril-Based Cataract Formation *In-vivo*?

**Published:** 2014

**Authors:** Mohammad Reza Nikbakht, Mohammad Reza Ashrafi-Kooshk, Morteza Jaafari, Moosa Ghasemi, Reza Khodarahmi

**Affiliations:** a *Faculty of Pharmacy, Kermanshah University of Medical Sciences, Kermanshah, Iran.*; b *Medical Biology Research Center, Kermanshah University of Medical Sciences, Kermanshah, Iran.*

**Keywords:** Timolol, Amyloid, Cataract, Crystallin

## Abstract

Timolol is a non-selective beta-adrenergic receptor antagonist administered for treating glaucoma, heart attacks and hypertension. In the present study, we set out to determine whether or not timolol can provoke cataract formation, thus the influence of timolol on the amyloid-type aggregation of crystallin was investigated. We then provided experimental evidence of crystallin aggregation and its induction by timolol using different spectroscopic measurements. Turbidimetric measurements as well as ThT fluorescence data indicated that timolol induce extent of crystallin amyloid formation. The kinetic of protein aggregation was also changed in presence of increasing concentrations of the drug suggesting that long-term drug administration may contribute to the development of cataract. Since the consequence of timolol-crystallin interaction has yet to be identified, additional data on it may help us to postpone amyloid cataract formation.

## Introduction

**Figure 1:**
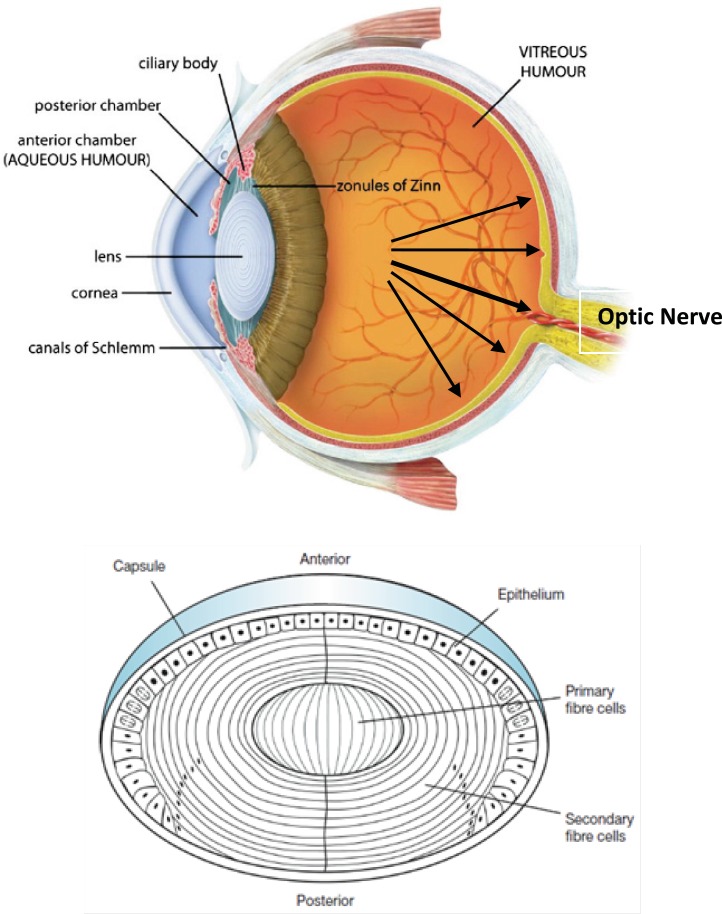
(**Top**) Sagittal section of the human eye. The anterior chamber between the cornea and lens contains the aqueous humour (AH) and the firm, gel-like vitreous humour (VH) is located behind the lens in the posterior part of the eye. In Glaucoma condition, elevated intraocular pressure (see black arrows inside eye) causes irreversible damage to optic nerve. (**Bottom**) Normal human lens morphology at birth. The lens is encapsulated by a basement membrane (lens capsule). The anterior of the lens contains a single layer of epithelial cells (lens epithelium), which divide and differentiate into secondary lens fibre cells (cortical fibres). The primary lens fibre cells (nuclear fibres) are formed during embryogenesis by differentiation of the posterior lens vesicle cells. The symmetrical and transparent structure of the lens is the result of an organized process of epithelial cell differentiation that includes elimination of large scattering organelles (dark dots) and the accumulation of lens crystallins to very high levels. Reproduced from ref. (2).

Glaucoma is an eye disease in which the optic nerve is damaged in a characteristic pattern (see Figure 1). This can permanently damage vision in the affected eye(s) and lead to blindness if left untreated. It is normally associated with increased fluid pressure in the eye (aqueous humour) (1, 2).

Intraocular pressure (IOP) can be lowered with medication. In addition to prostaglandin analogues (latanoprost (Xalatan), bimatoprost (Lumigan) and travoprost (Travatan)), α2-adrenergic agonists (brimonidine (Alphagan) and apraclonidine) and carbonic anhydrase inhibitors (dorzolamide (Trusopt), brinzolamide (Azopt), and acetazolamide (Diamox)), topical beta-adrenergic receptor antagonists, such as timolol (structure 1), levobunolol (Betagan), and betaxolol, decrease aqueous humor production by the ciliary body (3).

**Structure 1:**
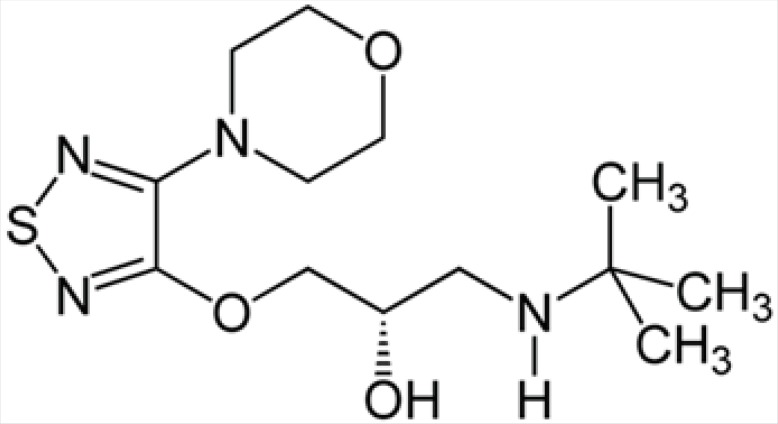
Chemical structure of timolol

**Figure 2:**
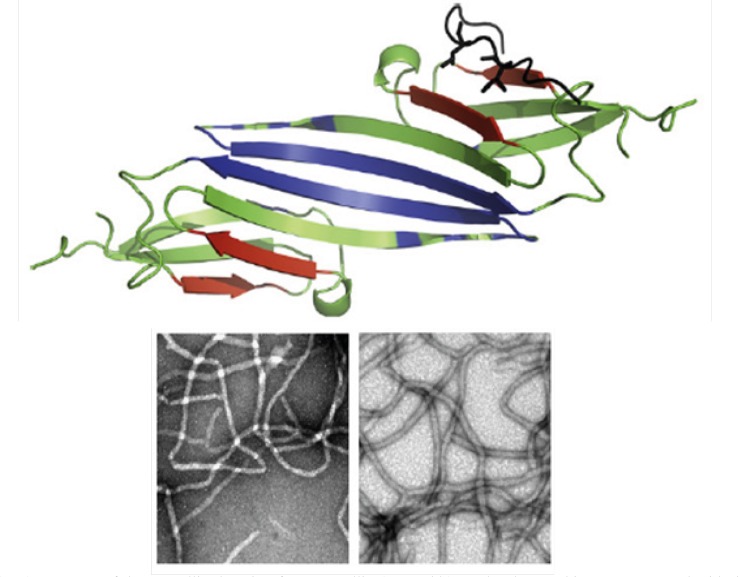
(**Top**) Structure of the crystallin domain of αB-crystallin (PDB 2klr). Molecular graphics were prepared with the SPDBV software (version 4.04) (available at: http://www.expasy.ch). All determined structures exhibit a 6- to 8-stranded β-sandwich structure (10). (**Bottom**) Fibril formation by αA- and αB-crystallins. Reproduced from ref. (7).

**Figure 3:**
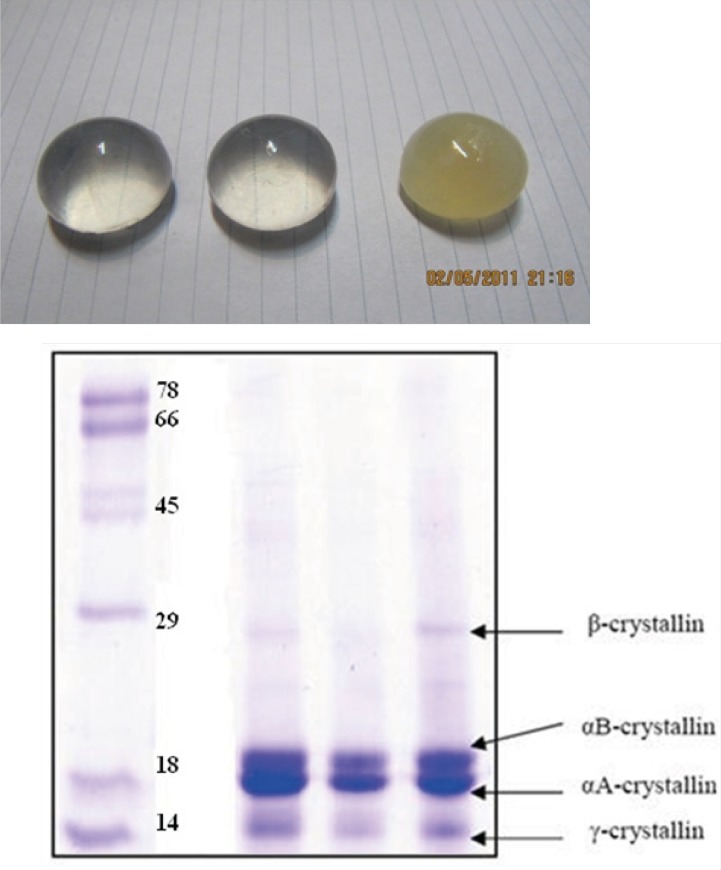
(**Top**) healthy and opaque bovine lens, collected in this study. (**Bottom**) SDS-PAGE pattern of purified α-crystallin (middle), crude extract (right) and molecular size markers (left). Further details are given in Experimental procedures

Cataract formation (a clouding of the normally clear eye lens, see Figures 1 and 3), on the other hand, affects distance vision and causes problems with glare, and is the most common cause of blindness worldwide. Cataracts are better known for affecting older people, but hereditary non-syndromic childhood cataracts occur at a frequency of about 1–6 cases per 10000 live births (4). Although such cataracts have been recognized for a century, the relationships between cataract etiology, morphology and underlying mechanisms are unclear. Crystallin proteins are responsible for maintaining the structure, refractive index and optical properties of lens fibre cells. The crystallins contribute to the transparency and refractive power of the lens by short-range interactions among themselves in a highly concentrated protein matrix (4). However, although crystallins mainly function as structural proteins in lens fibre cells, acrystallins can also function as chaperones and as anti-apoptotic proteins (5). Crystallins as well known molecular chaperones (Figure 2), have been also found to convert readily into amyloid fibrils, under slightly destabilizing conditions (6). These proteins are found in eye lenses as α-, β-, and γ-crystallins (7). Alpha-crystallin as major structural protein of eye lens is a mixture of two subunits, αA- and αB-crystallin with ratio of ~ 3:1, respectively (8). αB-crystallin is located in many parts of the body including the retina, heart, skin, brain, kidneys as well as lungs and associated, in significant quantities, with a diversity of neurodegenerative diseases, whereas αA-crystallin is also present to a much lesser extent in the spleen and thymus (9).

Two possible mechanisms, which are not mutually exclusive, may cause cataract (11). One mechanism is condensation phenomenon, whereby opacity results from loss of solubility of the crystallins. Some mutations dramatically lower the solubility of these proteins relative to that of the wild-type protein. In the second mechanism, cataract is seen as a conformational disorder where unfolding or destabilization of the crystallin proteins drives cataractogenesis. In the latter case, the researchers observed the ready assembly of the mutant α-crystallin into amyloid fibrils *in-vitro *under conditions where the wild-type protein remains soluble. Formation of amyloid deposits in the eye lens would potentially disturb the short range order of the crystallins and thus lead to lens opacity and cataract. Alpha (αA and αB)-crystallin is a molecular chaperone that maintains the optical properties of the lens and delays the onset scattering caused by aging-related protein aggregation. It has been found that the missense mutation R_116_H resulted in an altered size distribution, impaired packing of the secondary structures and modified quaternary structure with great hydrophobic exposure. It also appears that the aggregation of the mutant forms of crystallins upon stress may be responsible for the onset of cataract (12). Moreover, it has been reported that all three classes of wild-type crystallin proteins are capable of forming amyloid fibrils when subject to unfolding conditions *in-vitro *(see Figure 2). The inherent ability of the crystallins to convert into fibrils suggests that this process could contribute to the development of cataract with aging (11). Furthermore, considering protective effects of some molecular stabilizers such as carnosine (13) and inductive effect of timolol on the crystallin amyloid fibril formation, we can conclude that conserved α-crystallin chaperone activity may have a determinant role in restoring lens transparency.

In this work, we showed that timolol induce amyloid formation of lens α-crystallin. It is suggested that the resulting data (if established) can be useful in providing more safe intervention strategies against glaucoma. 

## Experimental

*Materials and equipments*

Congo Red (CR) and ThT were obtained from Sigma chemical company (St. Louis, MO, USA). Gel reagents and all other chemicals were the highest analytical grade of purity available and were used without further purifications. All solutions were prepared with double distilled water. Unless otherwise stated, all solutions were made in 100 mM potassium phosphate buffer (pH 7.4). Appropriate vehicle controls were run in all experiments. A Perkin-Elmer spectrophotometer (model Lambda 25) was used for protein determination, CR binding analysis and turbidimetric measurements. 

*Protein purification/determination*

Groups of healthy bovine lenses were collected immediately postmortem from cows of less than two years in age, within a 1 month period and stored at -70 °C (see Figure 3). Alpha-crystallin was then purified, as described previously (14, 15). Additionally, SDS-polyacrylamide gel electrophoresis was used to confirm the purity of protein. The protein samples were loaded on a 12% slab gel under nonreducing conditions according to the method of Laemmli (16). Protein concentrations of α-crystallin were determined according to Lowry’s method (17). Standard curve was generated using BSA. 

*Formation of amyloid fibrils by bovine* *α-crystallin*

Bovine α-crystallin was dissolved at different concentrations in 0.1 M phosphate buffer, pH 7.4, and incubated at 60 °C with 1 M guanidine hydrochloride (GdnHCl) (7), on a thermostatic serological water bath for the specified durations while they were being gently stirred. For amyloid formation with TFE, bovine α-crystallin was dissolved at 1.0 mg/mL in 10% (v/v) TFE, 0.1 M phosphate buffer (pH 7.4), and incubated at 60 °C (7). For ThT-based kinetic fluorescence measurements, aliquots were taken from incubated protein sample at varying time intervals and placed on ice prior to assay. Also, for turbidimetric measurements, the protein samples, at various concentrations, were heated to 60 ºC in a 0.9 mL quartz cuvette for the specified durations, in the presence or absence of timolol, on a spectrophotometer equipped with temperature controller apparatus using a sample cell with path length of 1 cm. Immediately, the increase in the turbidity was recorded in the kinetic mode by measuring the absorbance at 400 nm as a function of time relative to the appropriate blank. Whenever needed, turbidity values were normalized through dividing them by the maximal limiting turbidity observed (6). The kinetic data were also fitted to the model described by Kurganov (18). 

*ThT fluorescence Analyses*

To investigate kinetically whether α-crystallin, after the heating in the presence of GdnHCl, was converted to amyloid-like fibrils, and to evaluate the timolol’s effects on this process, a ThT-based fluorimetric method was employed according to our previous published work (6). All fluorescence measurements were carried out in the ratio mode on a Perkin Elmer LS 45 luminescence spectrophotometer (USA) equipped with a 150 W xenon lamp at room temperature or as it is stated.

*Congo red binding assay*

Fresh solutions of Congo red (CR) in 100 mM potassium phosphate buffer, pH 7.6, were passed through a 0.22 µm filter immediately before use. The CR stock solutions (1 mM) were added to aliquots of pre-heated protein solutions to yield final dye of 20 µM and the samples vortexed for 15 s. The absorption spectrum of each sample was recorded over a range of 400 to 700 nm using 1-cm path length quartz cuvettes after incubation at room temperature for at least 10 min and corrected for contributions from buffer. The spectrum of CR alone was compared with that of CR solutions in the presence of protein. A red shift of the absorption band toward 540 nm and increase in the absorption intensity were together taken to be indicative of the formation of amyloid structure (6). All experiments were performed in duplicate.

*Protein* *modification with glyceraldehyde*

Crystallin solution (3 mg/mL) was incubated overnight at 37 °C in an aqueous solution of 7.5 mM glyceraldehyde buffered with 50 mM phosphate, pH 7.4. At the end of the incubation time, 100 μL of a 0.1 M NaBH_4_ solution in 0.1 N NaOH was added to the preincubated protein solution and the mixtures were further incubated for 1 h at 37 °C (final volume: 1 mL). After cooling down at room temperature, the resulting modified proteins were dialyzed against 50 mM phosphate buffer pH 7.4 for 24 h. To determine the extent of lysine modification, the free amino groups in the protein were measured using the TNBS method as described elsewhere (19). Also, SDS-PAGE analyses of modified protein samples were done to ensure that no detectable protein oligomerization occurred.

*ANS fluorescence and determination of PSH*

Changes in PSH can be monitored by the probe 1-anilinonaphtalen-8-sulfonate (ANS) (20). Titration of protein solutions in the presence of increasing concentrations of ANS provide information about difference in the ANS binding properties of native and modified crystallins by determining the F_max_ and *K*_d_^app ^of the protein-ANS complexes. F_max _is maximum fluorescence intensity at the saturated ANS concentration which indicates the number of surface hydrophobic sites of the protein. *K*_d_^app ^is the apparent dissociation constant for ANS. The assay solutions (0.1 mg/mL protein in the presence of various concentrations of ANS) were excited at 380 nm and emissions were recorded over range 400-600 nm (20). ANS was added from a stock solution (1 mM) to the final concentration range from 2 to 120 µM. The increase in fluorescence emission was recorded until no further increase in fluorescence was observed. The protein surface hydrophobicity of native and modified crystallins can be calculated from the following equation: (20)

$\mathrm{PSH}=F_{\max}/\left[\alpha -\mathrm{crystallin}\right]k_{d}^{\mathrm{app}}$

*Sequence and hydropathy profile analyses*

Similarity searches were carried out using BLAST P and FASTA services through the NCBI and EBI (www.ebi.ac.uk) servers, respectively. Amino acid sequences (FASTA format) of αA-crystallin and αB-crystallin were derived from swissprot (or PDB) databases. The calculation and analyses of hydropathy profiles/hydrophobicity scales of αA-crystallin/αB-crystallin were performed according to (21) with window size 9.

## Results and Discussion

*Aggregation kinetics of α-crystallin*

**Figure 4:**
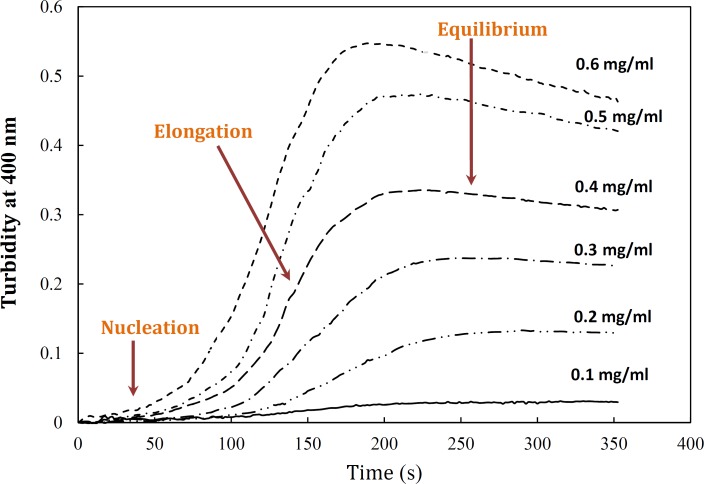
Effect of protein concentration on the kinetics of GdnHCl-induced α-crystallin aggregation at 60 °C. The final concentrations of the protein are shown within figure. Turbidity changes were normalized according to maximal change observed. The modified crystallin displayed no detectable aggregation (data not shown). Data shown are one representative example of three independent experiments. Further details are given in experimental procedures

Aggregation of α-crystallin was studied under the effect of different protein concentrations. Figure 4 demonstrates a gradual development of turbidity at 400 nm with increase in α-crystallin concentration. As shown by this figure, the duration of lag time was relatively large and reciprocally dependent on [P]_o_. As depicted in Figure 4, the amyloid formation process was found to obey the characteristic nucleation – elongation pattern, with three distinct phases: initial nucleation, elongation and equilibrium. 

**Figure 5:**
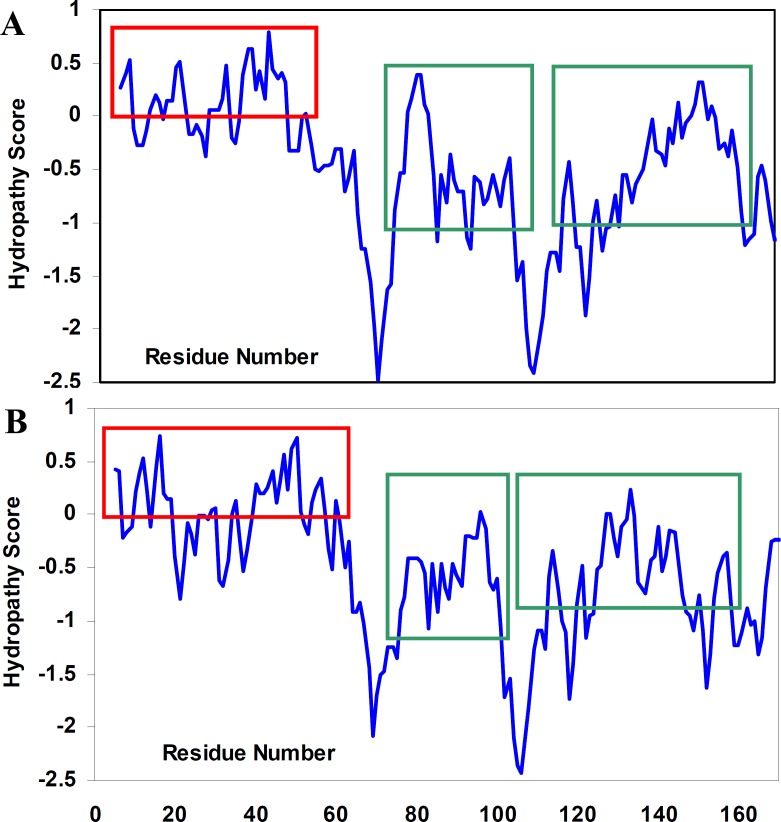
Hydropathy profiles of αA-crystallin (A) and αB-crystallin (B), calculated using the Roseman hydrophobicity scale (21) with window size 9. Note and compare the continuity and discontinuity in positive hydropathy scores, as indicated by red rectangulars. Also, note the homogeneity among hydropathy values in aggregation-prone segments (21) of crystallin sequences, as indicated by green rectangulars. See text for further details. For interpretation of the references to colour in this figure legend, the reader is referred to the web version of this article.

Surprisingly, the modified crystallin displayed no amyloid fibrillation under GdnHCl-induced α-crystallin aggregation conditions. It is believed that hydrophobic interactions are main driving forces of crystallin amyloid aggregation ((21), see also Figure 5). To re-test this possibility, PSH of the native and modified proteins were determined (20). As indicated in Table 1, PSH of the crystallin decreased after modification. These results are in full agreement with turbidimetric (Figure 1) and hydropathy (Figure 5) data confirming determinant role of hydrophobic interaction in crystalline amyloid formation. 

*Theoretical analyses of hydropathy profiles*

To the best of our knowledge, the structural determinants of the formation of amyloid fibrils are not clear. We, therefore, tried not only to investigate hydropathy profile in α-crystallin. Based on hydropathy profiles (Figure 5), apolar residues of crystallins consecutively have been distributed mainly through the aggregation-prone segments of polypeptide sequences. 

Furthermore, since the modified crystallin had no significant aggregation propensity, compared to the native protein, it was excluded from further studies.

**Table 1:** PSH data of the native and modified forms of α-crystallin

	*K*_a_ (µM)	Y-intercept	*K*_d_ (µM)	X-intercept	[Protein] (mg/ml)	PSH
* **Native**	**0.102±0.032**	**9.80±0.23**	**48.76±2.1**	**478.1±12.1**	**0.10**	**487.6±4.5**
* **Modified**	**0.158±0.036**	**6.33±0.016**	**38.32±1.4**	**242.7±8.3**	**0.10**	**383.2±6.9**

* Data shown are mean of three independent experiments and standard deviations were within 5% of the experimental values.

**Figure 6:**
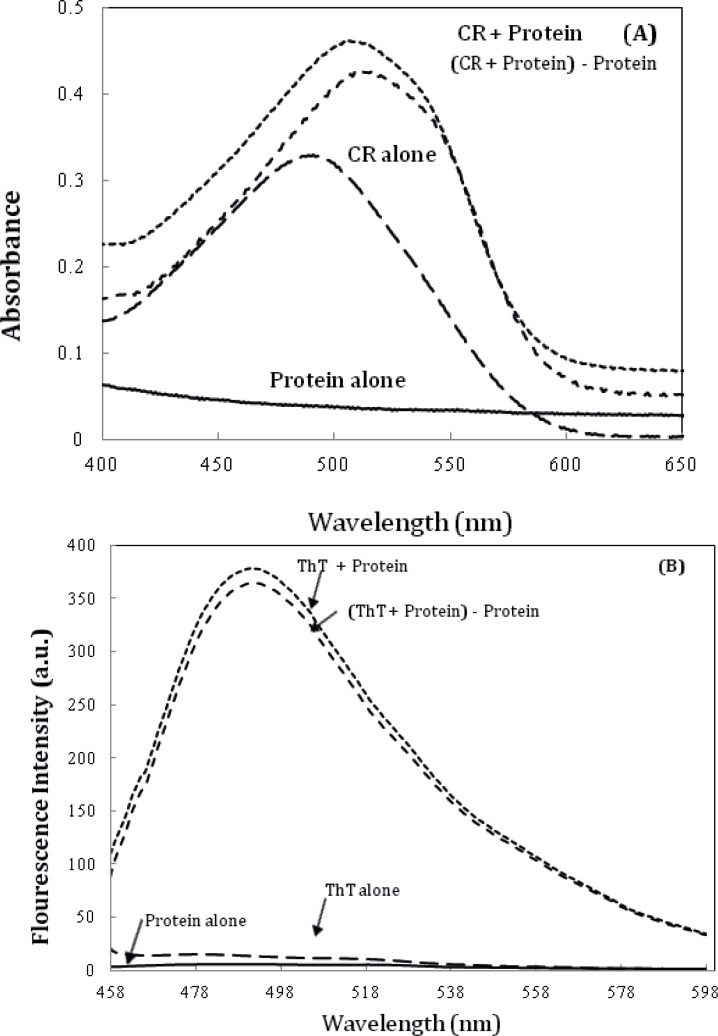
A) Alpha-crystallin amyloid fibrillation, as measured by monitoring changes in CR absorbance spectra. Crystallin, at 0.5 mg/mL concentration was incubated at 60 °C. The spectrum of CR alone was compared with that of CR solutions in the presence of 0.5 mg/mL Crystallin. (c) α-crystallin aggregation as indicated by ThT assay (the fluorescence intensity at 482 nm). The final protein and ThT concentrations were 0.05 mg/mL and 10 µM, respectively. Data shown are one representative example of three independent experiments. Further details are given in experimental procedures.

Further characterization of amyloid fibrils was performed using Congo red (CR) binding assay (6). The absorption spectra of CR on binding to the aggregates of the α-crystallin showed a red shift of the maximum absorbance of CR from ~ 490 nm to ~ 520 nm (Figure 6A), which is a characteristic of amyloid aggregation. The same observations were made using ThT fluorescence spectroscopy (Figure 6B) (6). 

**Figure 7:**
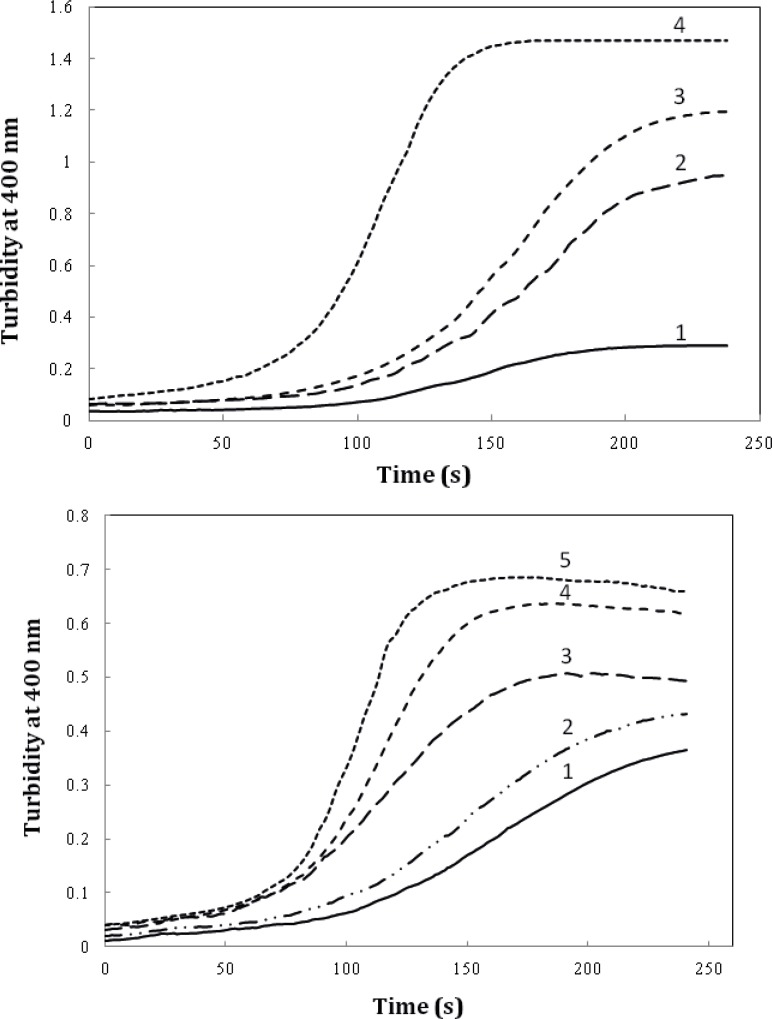
The effect of timolol on the GdnHCl-induced α-crystallin aggregation extent at 60 °C. (**A**) Protein (α-crystallin) and timolol concentrations were 0.6 (curves 1, 2), 0.8 (curves 3, 4) mg/mL and 5 μM, respectively. (**B**) After addition of α-crystallin (0.6 mg/mL) to 0.1 M sodium phosphate buffer (pH 7.4), containing 1, 2, 3, 4 and 5 µM of timolol, the absorption of each sample was measured at 400 nm with respect to the appropriate blank. Data shown are one representative example of three independent experiments

We also evaluated the effect of timolol on the extent of amyloid aggregation of crystallin. As evident from Figure 7A, timolol (5 μM) enhances the aggregation extent of the protein, at 0.6 mg/mL, by almost ~3-fold. Also, as indicated in Figure 7B, a significant and dose-dependent increase in the apparent aggregation extent of crystalline was observed in the presence of increasing concentrations of the drug. Further investigations are needed to disclose the mechanism of timolol-induced crystallin aggregation. Moreover, since in the present work, we have studied on Bovine α-crystallin, it is noteworthy that the human and bovine αA/αB-crystallins show more than 98% sequence identity, as revealed by amino acid sequence homology analyses.

**Figure 8:**
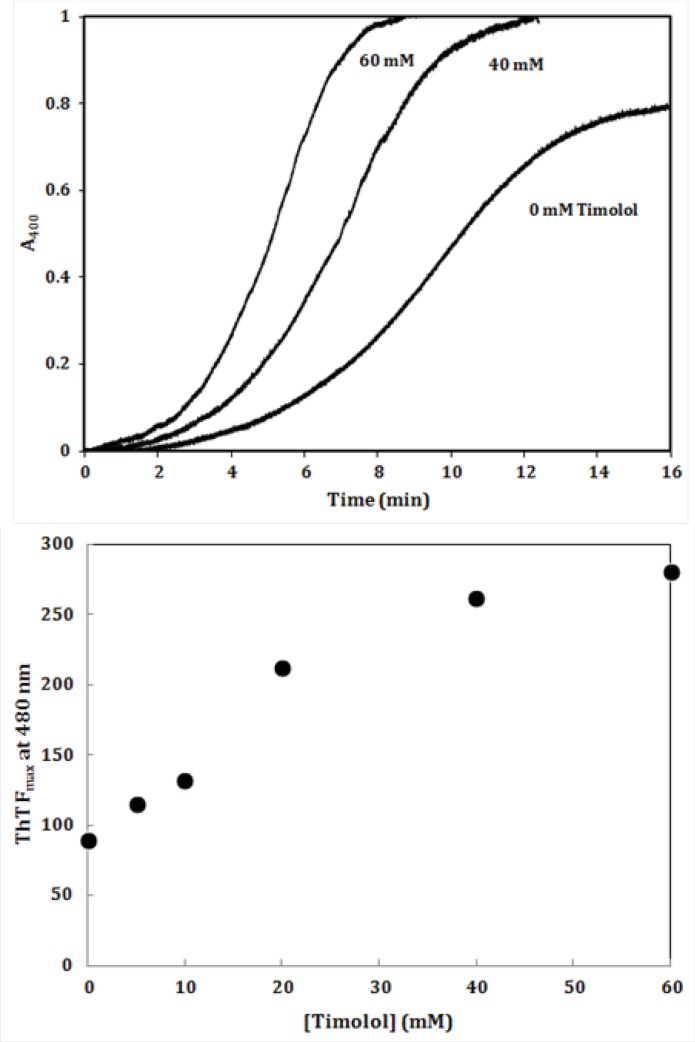
(**Top**) Effect of different concentrations of timolol on the aggregation kinetics (extent/rate) of α-crystallin. The mixed solutions of α-crystallin in the heating buffer containing various concentrations of timolol were heated with the result of the turbidity development, displaying different values on the absorbance at 400 nm and the ability of timolol to induce α-crystallin aggregation was documented. (**Bottom**) The relative emission intensity of ThT (at 480 nm) in the presence of timolol showed a significant increase, which is characteristic of enhanced amyloid aggregation and extended β-sheet (fibril) structures. Data shown are one representative example of three independent experiments and standard deviations were within 5% of the experimental values.

To further investigate the amyloidogenic property of timolol, heat-induced denaturation of α-crystallin was evaluated with (or without) timolol at different concentrations and the extent of aggregation was registered by monitoring the absorbance increment at 400 nm. As depicted in Figure 8, addition of timolol to the incubation mixture induced aggregate formation of α-crystallin. At 60 mM concentration of timolol, there was more than 50% induction of the α-crystallin aggregation. Incubation of timolol alone, at different concentrations tested, also, showed no change in turbidity. We then set out whether timolol induce amyloid aggregate formation or not. To test this possibility, additional characterization of amyloid fibrils was performed using ThT fluorescence assay. The relative emission intensity of ThT (at 480 nm) on binding to the aggregates of the α-crystallin in the presence of timolol showed a significant increase from ~ 100 to ~ 250 (Figure 8), which is a characteristic of enhanced amyloid aggregation, probably coinciding with the accelerated formation of extended β-sheet (fibril) structures. It is noteworthy that timolol has no effect on the emission spectrum of ThT.

**Figure 9:**
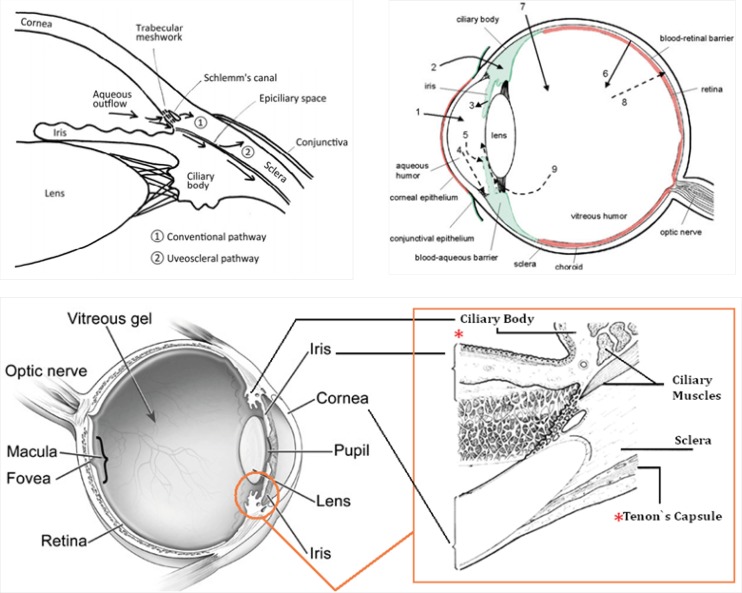
(**Top, left**) Schematic presentation of the two aqueous outflow pathways. Pathway is the conventional path through the TM and SC to the episcleral vein. Pathway is the uveoscleral pathway, in which aqueous travels through the iris root and ciliary muscle to the choroidal circulation or orbital tissues. (**Top, right**) The routes of drug kinetics. The numbers refer to following processes: 1) transcorneal permeation from the lacrimal fluid into the anterior chamber, 2) non-corneal drug permeation across the conjunctiva and sclera into the anterior uvea, 3) drug distribution from the blood stream via blood-aqueous barrier into the anterior chamber, 4) elimination of drug from the anterior chamber by the aqueous humor turnover to the trabecular meshwork and Sclemm's canal, 5) drug elimination from the aqueous humor into the systemic circulation across the blood-aqueous barrier, 6) drug distribution from the blood into the posterior eye across the blood-retina barrier, 7) intravitreal drug administration, 8) drug elimination from the vitreous via posterior route across the blood-retina barrier, and 9) drug elimination from the vitreous via anterior route to the posterior chamber. Taken from ref. (23). (**Bottom**) sites of action (Ciliary Body) and accumulation (Tenon’s Capsule) of timolol

Intraocular pressure (IOP) depends on the balance between the inflow and outflow of aqueous humor. In glaucoma patients, IOP is elevated because of an increase in outflow resistance. Two aqueous humor outflow pathways exist in the eye. Aqueous mainly flows through the trabecular meshwork (TM) and Schemm`s canal (SC) to the episcleral vein, but an auxiliary uveoscleral pathway through the iris root and ciliary muscle exists, with fluid leaving the eye through the choroidal circulation or orbital tissues (Figure 9, top, left). An increased resistance to flow in the main pathway, which carries 80% of total aqueous humor out of the eye, is predominantly responsible for elevated IOP in the many types of glaucoma in humans. In primary open-angle glaucoma (POAG), the most common type, increased outflow resistance occurs mainly in the juxtacanalicular (JCT) TM, the portion closest to SC, and in the endothelial-lined SC. 

Timolol is the most frequently prescribed (non selective) β-adrenoceptor antagonist; other agents in this class include bunalol, betaxolol and carvediol which lower IOP by attenuating aqueous humor formation and enhancing trabecular outflow (22). As a result of adrenoreceptor blockade in the ciliary body, decreased aqueous secretion, ultrafiltration, or both may occur. The most commonly reported side effects of timolol administration in people include local irritation and conjunctival hyperemia. Potential systemic risks associated with topical timolol use are related to systemic β-adrenoreceptor blockade. Cardiac arrhythmias, heart block, and bradycardia can occur with β_1_-blockade, whereas pulmonary effects, such as bronchospasm and airway obstruction, can result from β_2_-blockade. Beta-blocking agents are not recommended for first-line glaucoma therapy in patients with cardiovascular compromise or a history of pulmonary disease.

It is noteworthy that prescribed drugs (such as timolol) have several routes of administration and elimination from the eye which have been shown schematically in Figure 9. (top, right) (23).

Taking above statements into account and since timolol needs to penetrate within intraocular cavity to interact with its target site (ciliary body), there is this possibility that the drug diffuse into eye lens (neighbor of Ciliary Body, see Figure 9, top, left and bottom) and interact with c*rystallins *that compose over 90% of the protein within the *lens. *Furthermore, periocular accumulation of timolol in glaucoma patients under long-term therapy has been previously reported so that more than 1 mg of the β-adrenergic antagonist was estimated to be present periocularly within the intact Tenon’s Capsule (24). Thus, the drug accumulation within lens may be anticipated. At these conditions and as documented in this study, timolol-crystallin interaction may promote/trigger *in-vivo* (amyloid) aggregation of α-crystallins.

In summary, since timolol and other anti-glaucoma drugs are widely administered worldwide (25), there is the possibility that prolonged treatment of enhanced IOP play an undesired causative role in triggering onset of lens opacity, especially, in susceptible individuals carrying mutant forms of crystallin proteins. Also, the resulting data (if established) may be useful in providing mechanistic insights to develop potential curative and/or preventive strategies *in-vivo *against amyloid-related cataracts. This type of aggregation-based challenges like *in-vitro* protein aggregation (a serious problem in the formulation of therapeutic proteins (26)) needs more attention.
